# Validation of the easyscreen flavivirus dengue alphavirus detection kit based on 3base amplification technology and its application to the 2016/17 Vanuatu dengue outbreak

**DOI:** 10.1371/journal.pone.0227550

**Published:** 2020-01-17

**Authors:** Crystal Garae, Kalkoa Kalo, George Junior Pakoa, Rohan Baker, Phill Isaacs, Douglas Spencer Millar

**Affiliations:** 1 Vila Central Hospital, Port Vila, Vanuatu; 2 Genetic Signatures, Sydney, Australia; Faculty of Science, Ain Shams University (ASU), EGYPT

## Abstract

**Background:**

The family flaviviridae and alphaviridae contain a diverse group of pathogens that cause significant morbidity and mortality worldwide. Diagnosis of the virus responsible for disease is essential to ensure patients receive appropriate clinical management. Very few real-time RT-PCR based assays are able to detect the presence of all members of these families using a single primer and probe set. We have developed a novel chemistry, 3base, which simplifies the viral nucleic acids allowing the design of RT-PCR assays capable of pan-family identification.

**Methodology/Principal finding:**

Synthetic constructs, viral nucleic acids, intact viral particles and characterised reference materials were used to determine the specificity and sensitivity of the assays. Synthetic constructs demonstrated the sensitivities of the pan-flavivirus detection component were in the range of 13 copies per PCR. The pan-alphavirus assay had a sensitivity range of 10–25 copies per reaction depending on the viral strain. Lower limit of detection studies using whole virus particles demonstrated that sensitivity for assays was in the range of 1–2 copies per reaction. No cross reactivity was observed with a number of commonly encountered viral strains. Proficiency panels showed 100% concordance with the expected results and the assays performed as well as, if not better than, other assays used in laboratories worldwide. After initial assay validation the pan-viral assays were then tested during the 2016–2017 Vanuatu dengue-2 outbreak. Positive results were detected in 116 positives from a total of 187 suspected dengue samples.

**Conclusions/Significance:**

The pan-viral screening assays described here utilise a novel 3base technology and are shown to provide a sensitive and specific method to screen and thereafter speciate flavi- and/or alpha- viruses in clinical samples. The assays performed well in an outbreak situation and can be used to detect positive clinical samples containing any flavivirus or alphavirus in approximately 3 hours 30 minutes.

## Introduction

Flavivirus is a genus of the family *Flaviviridae* that contains a large number of viral agents capable of causing encephalitis and jaundice [1]. Most flaviviruses are arboviruses and transmitted to the human population by a bite from infected mosquitoes or ticks. Flaviviruses typically contain a positive sense single-stranded RNA genome of approximately 10-11kb in length. The genome encodes 3 structural proteins (Capsid, prM, and Envelope) and 8 non-structural proteins (NS1, NS2A, NS2B, NS3, NS4A, NS4B, NS5 and NS5B) [2]. The viruses are enveloped with a diameter of around 50nm, and appear icosahedral or spherical when observed under the electron microscope [3]. Individual members such as dengue (DENV), yellow-fever virus (YFV), Japanese encephalitis virus (JEV), tick-borne encephalitis virus (TBEV) and West Nile virus (WNV) cause significant morbidity and mortality worldwide.

DENV is a major public health concern on a global scale with an estimated 400 million infections and 100 million clinical cases in 2010. Most of these patients will carry the disease asymptomatically. However, around 5% of infected individuals will progress to severe dengue, an illness characterized by plasma leakage leading to hypovolemic shock, hemorrhage, and potentially death. The case-fatality rate for individuals with severe dengue can be as high as 10% if untreated, or 0.1% with appropriate clinical management [4].

Alphaviruses are a diverse group of viruses that are classified as belonging to the group IV Togaviridae family of viruses [5]. There are over thirty members in the alphavirus group that are able to infect a wide range of vertebrates including humans, rodents, fish, birds, and horses. At the genomic level alphaviruses consist of a positive sense, single-stranded RNA genome 11 to 12kb in length with a 5’ cap, and 3’ poly-A tail. Alphavirus particles are enveloped, have a size of around 70 nm in diameter under the electron microscope and appear to be spherical with a 40 nm isometric nucleocapsid. Like flaviviruses the main mode of transmission to the human population is via bites from infected mosquitoes. Notable viruses that infect the human population include chikungunya (CHIKV), Barmah Forest virus (BFV), Mayaro virus (MAYV) [6], O'nyong'nyong virus (ONNV) [7], Ross River virus (RRV) [8], Una virus [9] and Tonate virus [10].

Epidemics of flavivirus and alphavirus occur globally on an annual basis with different degrees of severity. Table 1 shows a small selection of recent flavivirus/alphavirus outbreaks worldwide.

**Table 1:** Examples of recent flavivirus/alphavirus outbreaks worldwide.

Year	Location	Virus	Cases	Death rate complications	Reference
**2011**	Uganda	YFV	181	45 (24.9%)	[11]
**2012**	Texas, USA	WNV	1,868	89 (5%)	[12]
**2015**	India	DENV	99,913	200 (0.2%)	[13]
**2016–2017**	Latin America Caribean	ZIKV	217,000	3,400 associated congenital syndrome	[14]
**2016**	Taiwan	DENV	22,777	182 (0.8%)	[15]
**2016**	Odisha	JEV	336	103 (30.6%)	[16]
**2017**	Bangladesh	CHIKV	13,176	N/A	[17]
**2017**	Australia	RRV	>1,000	N/A	[18]

The global distribution and severity of flavivirus and alphavirus infection requires accurate surveillance tools and timely diagnosis to ensure infected patients obtain the best medical treatment options and alert authorities to possible outbreaks of disease.

The most accurate method to diagnose viral agents is real time Polymerase Chain Reaction (RT-PCR). Primer and probe sequences complementary to the viral RNA are designed and cycled through a series of steps with positive samples seen as amplification curves on a RT-PCR instrument. This process can be completed in less than 1 hour, which significantly assists in patient management.

However, members of the flavivirus and alphavirus families are quite heterogeneous at the RNA level, therefore it can be difficult to design a single set of primers and probe sequences that can detect each of the families at the genus and species level. An example of this is DENV that contains four serotypes, each being quite diverse at the genomic level [19]. Like most current dengue RT-PCR assays, the CDC DENV-1-4 RT-PCR Assay detects serotypes 1–4 using an individual primer pair and probe for each type [20]. Assays that can universally detect all DENV serotypes have been described but these assays still employ more than 2 primers to detect all subtypes [21].

**Fig 1:**
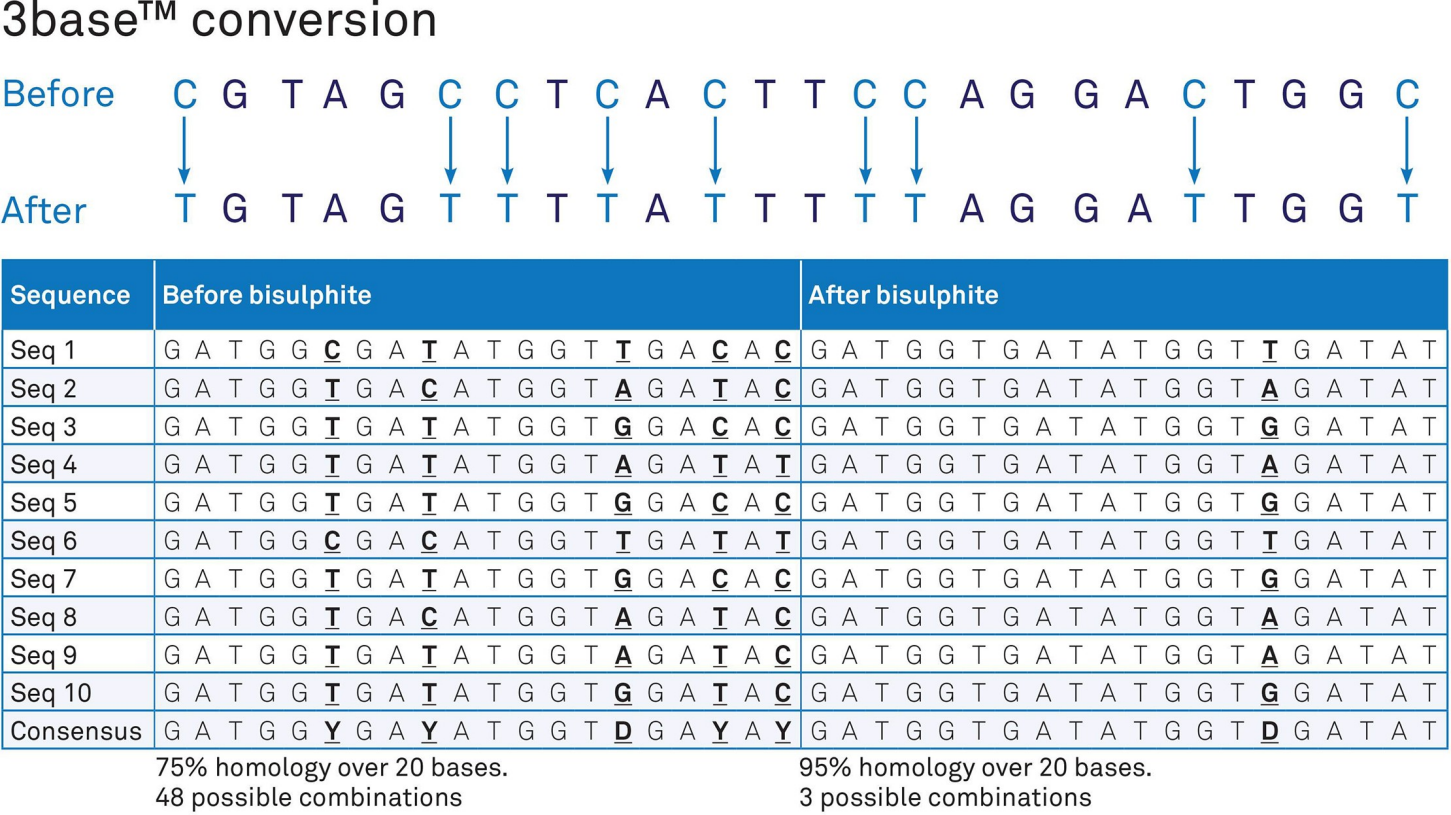
Example of the 3base mechanism. The example sequences below show the increase in homology from 75% (“Before”) to 95% (“After”) via the 3base conversion where all C bases are detected as T bases.

In order to simplify and improve the detection of alphaviruses and flaviviruses in clinical samples, we developed a commercially available 3base assay that is able to detect the presence of the target alphavirus or flavivirus using a single primer and probe set for each type. 3base assays use chemical modification to reduce the complexity of genomes from 4 to 3 base, which enable screening primers and probes with fewer mismatches to be developed so that bias in amplification efficiency across species is greatly reduced. (Fig 1).

**Fig 2:**
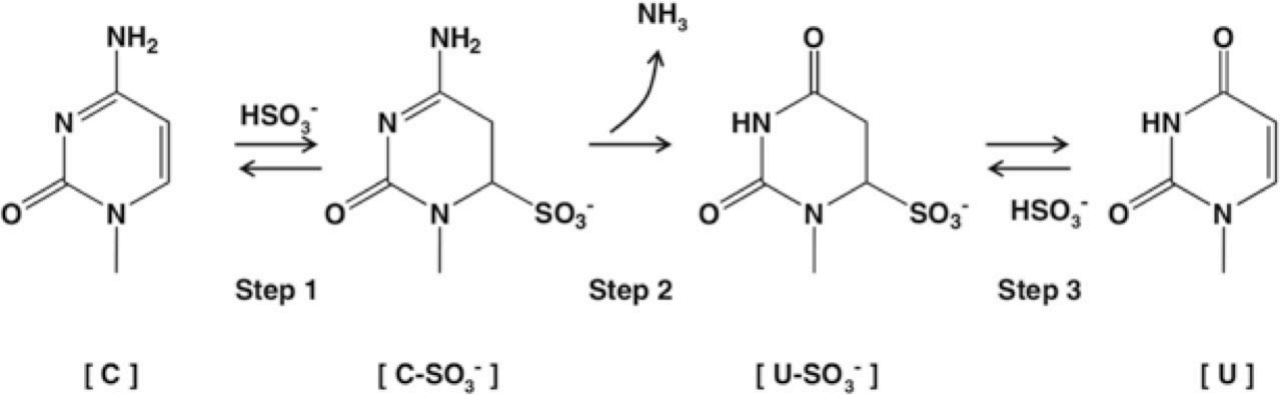
A diagram showing the 3base conversion process at the nucleotide level. The bisulphite reaction deaminates cytosine residues in nucleic acids to a uracil sulphonate. After treatment the base is then desulphonated to yield uracil. PCR amplification then converts this to a thymine, which results in essentially a 3base genome (A, G and T).

The 3base protocol (Fig 2) deaminates all cytosine residues in nucleic acids to a uracil intermediate [22]. This process makes closely related species more similar at the genomic level. This novel method ultimately means that primers and probe sets can be designed that have fewer mismatches and are able to hybridise to previously heterogeneous target regions with higher efficiency, thus improving PCR amplification of species that contain large numbers of individual pathogens.

The modification process of the genomic nucleic acids to a 3base form does not sacrifice specificity and individual typing primers can be constructed to detect the exact organism responsible for disease.

The method has been used to successfully detect the presence of high risk HPV in clinical samples [23] and the presence of pathogens, including Norovirus, in patients with gastrointestinal disease [24].

We have utilised the method to produce pan-species assays for the detection of all flavivirus, alphavirus and dengue serotypes 1–4 and successfully applied these assays to screen samples in the 2016/17 Vanuatu dengue outbreak.

## Methods

### Ethics statement

The interim ethics committee comprising the following members of the Ministry of Health; Mr. George Taleo the Director General of Health, Mr. Len Tarivonda the Director of Public Health, Mr. Russel Tamata the Director of Hospital and Curative Health and Dr. Posikai Samuel Tapo the Director of Cooperative Services, Planning and Policy approved the study. All adults provided verbal consent either for themselves or for their children to be included in the study.

### Synthetic constructs

Synthetic DNAs for the following viruses were synthesised (Sigma, Castle Hill, Australia) to determine the specificity and sensitivity of the individual PCR component of the assays. **Flaviviruses**; DENV-1, DENV-2, DENV-3, DENV-4, WNV, ZIKV, YFV, TBEV and MVEV. **Aphaviruses**; CHIKV, BFV, RRV, Eastern equine encephalitis virus (EEEV), Western equine encephalitis virus (WEEV), Venezuelan equine encephalitis virus (VEEV), Middleburg virus (MV) and Ndumu virus (NV).

These DNAs consist of single stranded 110bp DNA sequences that contain the identical primer and probe binding sites that are present in the viral nucleic acids of the target virus. In addition, the size of the amplicons are the same as those amplified in the viral nucleic acids to ensure results generated are consistent with what would occur in the native virus.

### Commercially available whole genomic RNAs and virus particles

Table 2 lists the nucleic acids or whole organisms used to determine sensitivity and specificity of the pan flavivirus, alphavirus and dengue assays.

**Table 2:** Materials used in sensitivity/specificity studies.

Flavivirus/Alphavirus reference materials			Nucleic acids used in cross-reactivity studies		
**Virus**	Supplier	Catalogue#	**Virus**	Supplier	Catalogue#
**CHIKV**	Vircell	MBC099	**adenovirus**	Vircell	MBC001
**CHIKV**	Zeptometrix	NATCHIKV-ST	**BK**	Vircell	MBC051
**DENV1-4, CHIKV, ZIKV Amplirun total**	Vircell	MBT023	**coronavirus**	Vircell	MBC090
**DENV-1**	Vircell	MBC055	**CMV**	Vircell	MBC016
**DENV-2**	Vircell	MBC056	**EBV**	Vircell	MBC065
**DENV-3**	Vircell	MBC057	**enterovirus 68**	Vircell	MBC125
**DENV-4**	Vircell	MBC058	**enterovirus 71**	Vircell	MBC019
**EEEV**	Vircell	MBC097	**HSV-1**	Vircell	MBC023
**RRV**	Vircell	MBC130	**HSV-2**	Vircell	MBC024
**SLEV**	Vircell	MBC101	**HHV-6**	Vircell	MBC025
**TBEV**	Vircell	MBC045	**influenza H1**	Vircell	MBC028
**VEEV**	Vircell	MBC096	**influenza H3**	Vircell	MBC029
**WEEV**	Vircell	MBC098	**influenza B**	Vircell	MBC03
**WNV**	Vircell	MBC069	**parainfluenza-1**	Vircell	MBC037
**WNV**	Zeptometrix	NATWNV-0005	**parainfluenza-2**	Vircell	MBC038
**YFV**	Vircell	MBC100	**parainfluenza-3**	Vircell	MBC039
**ZIKV (Asian)**	Vircell	MBC103	**parainfluenza-4A**	Vircell	MBC050
**ZIKV**	Vircell	MBC072	**rhinovirus**	Vircell	MBC091
**ZIKV**	Zeptometrix	NATZIKV-ST	**RSV (subtype A)**	Vircell	MBC041
**ZIKV range validation**	Zeptometrix	NATZIKV-RV	**RSV (subtype B)**	Vircell	MBC083
			**VZV**	Vircell	MBC048

### Validation panels

In addition, quality assurance panels for DENV, CHIKV and ZIKV were obtained from QCMD (Quality Controls for Molecular Diagnostics, Glasgow, Scotland). These panels contain inactivated whole viral particles in a range of different media.

### Extraction of synthetic constructs

Serial dilutions of these controls were converted to a 3base form using the *EasyScreen* SP001 Sample Processing Kit, SP001 (Genetic Signatures, Sydney, Australia) according to the manufacturer’s instructions.

### Assay lower limit of detection study

Nucleic acids and viral particles were reconstituted according to the manufacturers instruction. For lower limit of detection (LLOD) studies 100μl of the Vircell total run control (whole viral particles which are provided with a certificate specifying their copy number) and 10μl of Vircell DENV-1, DENV-2, DENV-3, DENV-4 and chikungunya RNA samples (again provided with a certificate specifying their copy number) were individually two fold serially diluted in a total volume of 100 μL molecular grade water (G-biosciences, St Louis, USA) or human serum to mimic actual clinical samples (H4522, Sigma, Sydney, Australia).

Samples were then converted using the *EasyScreen* Sample Processing Kit, SP001 (Genetic Signatures, Sydney, Australia) according to the manufacturer’s instructions. Nucleic acid purification was then completed using the GS-mini automated nucleic extraction platform using the *EasyScreen* Sample Processing Kit, SP006 (Genetic Signatures, Sydney, Australia) according to the manufacturer’s instructions.

After sample purification ten PCR replicates from each dilution were performed and the LLOD determined as the lowest dilution in which all ten PCR replicates were positive in the assay. The quantified copy number provided by the manufacturer (Vircell) was relied upon to determine copies per PCR.

### Preparation of contrived urine samples

Contrived urine samples were generated by spiking whole viral particles into 100 μL of freshly collected negative urine or water. Five μL of the Extraction Process Control (EPC) was then added to the sample prior to conversion. The EPC consists of intact MS2 phage and is added to the sample to control for proper sample conversion, reverse transcription and to monitor any PCR inhibition. Samples were then converted using the *EasyScreen* Sample Processing Kit, SP001 (Genetic Signatures, Sydney, Australia) according to the manufacturer’s instructions.

Nucleic acid purification was then completed using the GS-mini automated nucleic extraction platform using the *EasyScreen* Sample Processing Kit, SP006 (Genetic Signatures, Sydney, Australia) according to the manufacturer’s instructions.

### Extraction of potential cross reactants

In order to maximise the concentration of potential cross-reacting viruses (Table 2) in the sample eluate, 15 μL of Amplirun controls were extracted along with 5 μL of the EPC. A positive control (Vircell Amplirun DENV/ZIKV/CHIKV total run control) was included in the run to confirm all components of the assay were working optimally. The nucleic acids in the Amplirun control are provided at a concentration of between 10,000–20,000 copies per μL to give a total concentration in the eluate of 150,000–300,000 copies of viral nucleic acids. Nucleic acid conversion and extraction was carried out as above.

### Extraction of validation panels

Validation panels were handled as detailed in the by the manufacturer and 100 μL of material along with 5 μL of EPC was converted to a 3base form using the *EasyScreen* Sample Processing Kit, SP001 (Genetic Signatures, Sydney, Australia) according to the manufacturer’s instructions. After bisulphite conversion the samples were extracted on the GS-mini automated nucleic extraction platform using the *EasyScreen* Sample Processing Kit, SP006 (Genetic Signatures, Sydney, Australia) again according to the manufacturer’s instructions.

### Clinical samples

Serum or plasma samples from patient with suspected DENV infection were collected at Port Vila Central Hospital, Efate, Vanuatu between December 2016 to March 2017. 100 μL of material along with 5 μL of EPC was processed using the *EasyScreen* Sample Processing Kit, SP001 (Genetic Signatures, Sydney, Australia) according to the manufacturer’s instructions. After bisulphite conversion the samples were extracted on the GS-mini automated nucleic extraction platform using the *EasyScreen* Sample Processing Kit, SP006 (Genetic Signatures, Sydney, Australia) according to the manufacturer’s instructions.

### Primers and probes

The following primer and probe sets are available from Genetic Signatures: pan-flavivirus, pan-alphavirus and pan-dengue (FA001), Zika virus (FA002-A), West Nile virus (FA002-B), Yellow Fever Virus (FA002-C), Tick-borne encephalitis virus (FA002-D), St Louis encephalitis virus (FA002-E), Murray Valley encephalitis virus (FA002-F), dengue-1 (FA003-A), dengue-2 (FA003-B), dengue-3 (FA003-C), dengue-4 (FA003-D), chikungunya (FA004-A), Ross River virus (FA004-B), Barmah Forest virus (FA004-C), Eastern equine encephalitis virus (FA004-C), Western equine encephalitis virus (FA004-D) and Venezuelan equine encephalitis virus (FA004-E).

### Real-time reverse transcription PCR

Following sample extraction and elution in 50 μL, 2–4 μL of converted nucleic acids material were added to the combined PCR mastermix and components using the *EasyScreen* Flavivirus Dengue Alphavirus Detection Kit. (Genetic Signatures, Sydney, Australia). Thermocycling consisted of 1 cycle of reverse transcription at 42°C for 30 minutes, followed by Taq polymerase activation at 95°C for 15 minutes, then 50 cycles of amplification at 95°C for 2 seconds, 55°C for 15 seconds (data collection), 60°C for 15 seconds and 65°C for 15 seconds. PCR amplification was carried out using either the Biorad CFX96/384 (Biorad, Hercules, USA) or the MIC (Biomolecular Systems, Sydney, Australia) platforms.

### Statistical analysis

Disease incidence for any given island was compared to the average incidence for the whole country, using the z-score test for two population proportions available at https://www.socscistatistics.com/tests/ztest/default2.aspx. *P* values < 0.05 were considered statistically significant.

## Results

**Fig 3:**
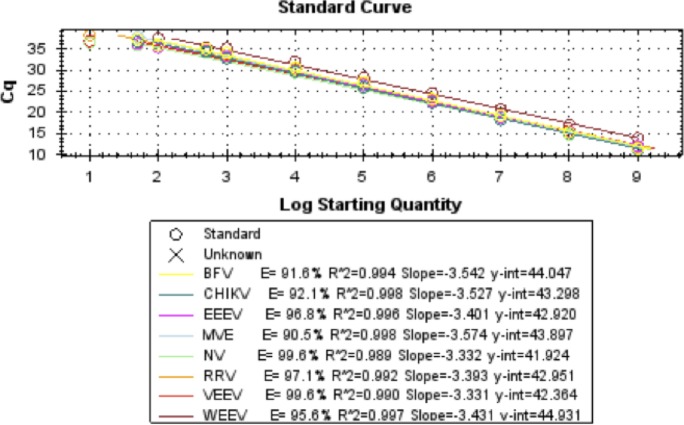
Results obtained using the pan-alphavirus assay (Cycle threshold (Ct) from a single PCR replicate). The standard curve was plotted using the log10 concentration versus Ct value.

**Fig 4:**
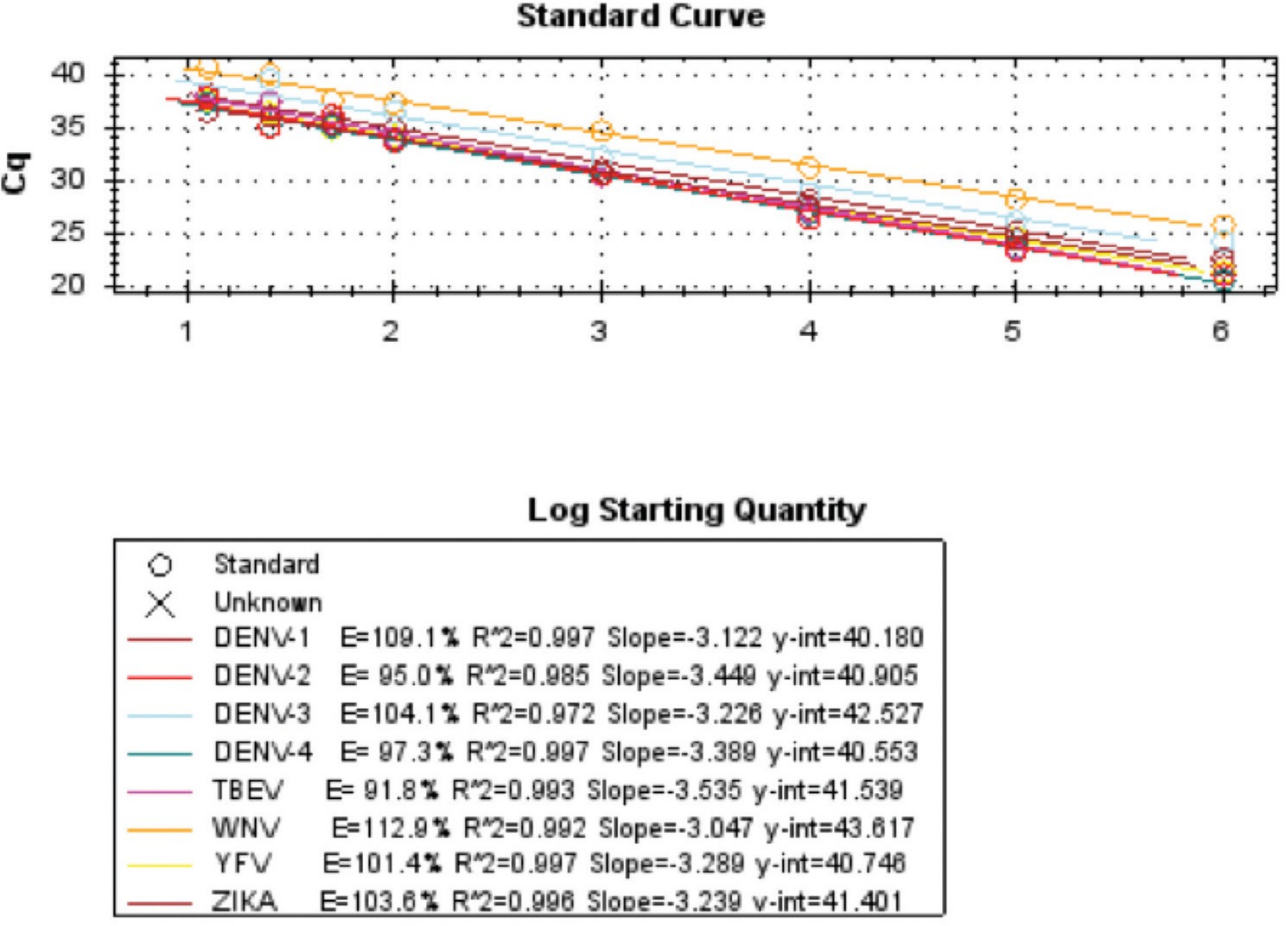
Results obtained using the pan-flavivirus assay (Cycle threshold (Ct) from a single PCR replicate). The standard curve was plotted using the log10 concentration versus Ct value.

Synthetic constructs were used to assess the performance of the pan-flavivirus, pan-alphavirus and pan-dengue assays and determine if the pan-viral assays were able to detect a wide selection of both flavi- and alphaviruses. Fig 3 shows the results obtained using the pan-alphavirus assay while Fig 4 shows the results obtained with the pan-flavivirus assay.

To determine the feasibility of using urine as a primary sample, fresh urine was spiked with both NATtrol Zika Stock (Qualitative) (Manufacturer, CATALOG# NATZIKV-ST) and NATtrol Zika Virus Range Verification Panel (Manufacturer, CATALOG# NATZIKV-RV). The whole cells and control materials were processed as previously described and compared to the same samples spiked directly into water. No significant delays between the urine samples and water controls were observed indicating that urine is not inhibitory in the sample processing method and suitable to use as a primary patient sample.

In order to determine the LLOD of the assays, the Vircell total run control (whole inactivated viruses containing a mixture of DENV-1, DENV-2, DENV-3, DENV-4, CHIKV and ZIKV in a plasma matrix) and genomic RNA from DENV-1, DENV-2, DENV-3, DENV-4 and CHIKV (Vircell, Valencia, Spain) and were used. Samples were diluted in human serum to simulate clinical samples and processed as previously described followed by amplification in ten individual replicates. LLOD was defined as the lowest dilution in which all ten PCR replicates gave a positive signal. The LLOD for the flavivirus assay was found to be 0.63 copies per PCR, 2.18 copies per PCR for the pan-dengue assay and 1.25 copies for the pan-alphavirus assay using whole virus particles. Interestingly, the whole viral samples performed better than the purified RNAs when diluted in human serum (Table 3).

**Table 3:** LLOD for each viral target of the assay.

LLOD Studies copies per reaction (10 replicates)			
Virus	Flavivirus	Alphavirus	Dengue
Run Control*	0.63	1.25	2.18
**DENV1**	14	N/A	56.25
**DENV2**	81.25	N/A	81.25
**DENV3**	39	N/A	39
**DENV4**	56.25	N/A	112.5
**CHIKV**	N/A	20.3	N/A

*Whole inactivated virus material in a plasma matrix

No cross reactivity was observed when testing the assay against 150,000–300,000 copies of viral nucleic acids introduced into the PCR reaction using the targets listed in Table 2. In all cases the EPC was positive, as was the positive control, indicting that the lack of amplification was not due to failure of any component of the assay or the presence of PCR inhibitors.

To further demonstrate the utility of the pan-species assays, QCMD panels were tested. The results obtained were in 100% agreement with the expected results when decoded (Table 4). Bold samples represent “educational samples”, which may or may not be detected by all participating laboratories as these samples mimic a clinical sample at the LLOD of most assays.

**Table 4:** Results of the 2016–2018 QCMD proficiency panels.

	QCMD proficiency panel data 2016–2018					
	Dengue panels		Zika panels		Chikungunya panels	
Year	Core	Educational	Core	Educational	Core	Educational
**2016**	8	2 **(83.8%, 79.4%)**	10	0	ND	ND
**2017**	7	3 **(86.1%, 80.6%, 56.9%)**	8	0	8	2 **(82.4%, 52.9%)**
**2018**	9	1 **(79.4%)**	8	0	9	1 **(66.2%)**

Core proficiency samples are reviewed by the QCMD Scientific Expert(s). This is on the basis of scientific information, clinical relevance, current literature and, where appropriate, professional clinical guidelines. Participating laboratories are expected to report core proficiency samples correctly within the EQA challenge. **Percentage Correct (All):** Percentage of datasets (%) reporting the correct qualitative result. ND, not done.

### Clinical testing

Prior to testing samples, a small study was performed to determine if alternative samples types such as mouth swabs or urine samples from patients suspected of DENV infection could be used as a less invasive alternative sample type. Four matched urine, plasma and mouth swab samples were taken and these extracted using standard procedures as described. Although DENV RNA could be detected in all three sample types, a significant Ct lag was observed for urine and mouth swab compared to plasma (S1 Fig). The data indicated there was approximately 100,000 fold more virus in serum/plasma samples compared to other matrix, further demonstrating that serum/plasma are the preferred test sample types. These data also agree with a previous study using viral RNA and NS1 antigen assays [25].

A total of 187 samples were tested (Table 5) during the 2016–2017 Vanuatu dengue-2 outbreak. The original testing algorithm consisted of screening each sample for pan-flavivirus, pan-alphavirus and pan-dengue only. During the first day of testing, pan-flavivirus/pan-dengue positive samples were subsequently typed with dengue typing primers that showed the serotype responsible for the current outbreak was DENV-2. In order to provide a more rapid diagnostic, DENV-2 primers were included at the request of the hospital in the initial screening assay to negate the need to perform subsequent reflex testing on the samples but still having the ability to detect new viruses if they emerged.

**Table 5:** Results of clinical sample obtained during the Vanuatu outbreak.

	Number of sample	% Positive
**pan-flavivirus**	116	62
**pan-alphavirus**	0	0
**pan-dengue**	123	66
**DENV-2**	116	62
**dengue not typed**	7*	3.2
**Negative**	64	34

* All 7 not typed pan-Dengue positive samples were extremely weak with Ct values>40.

**Fig 5:**
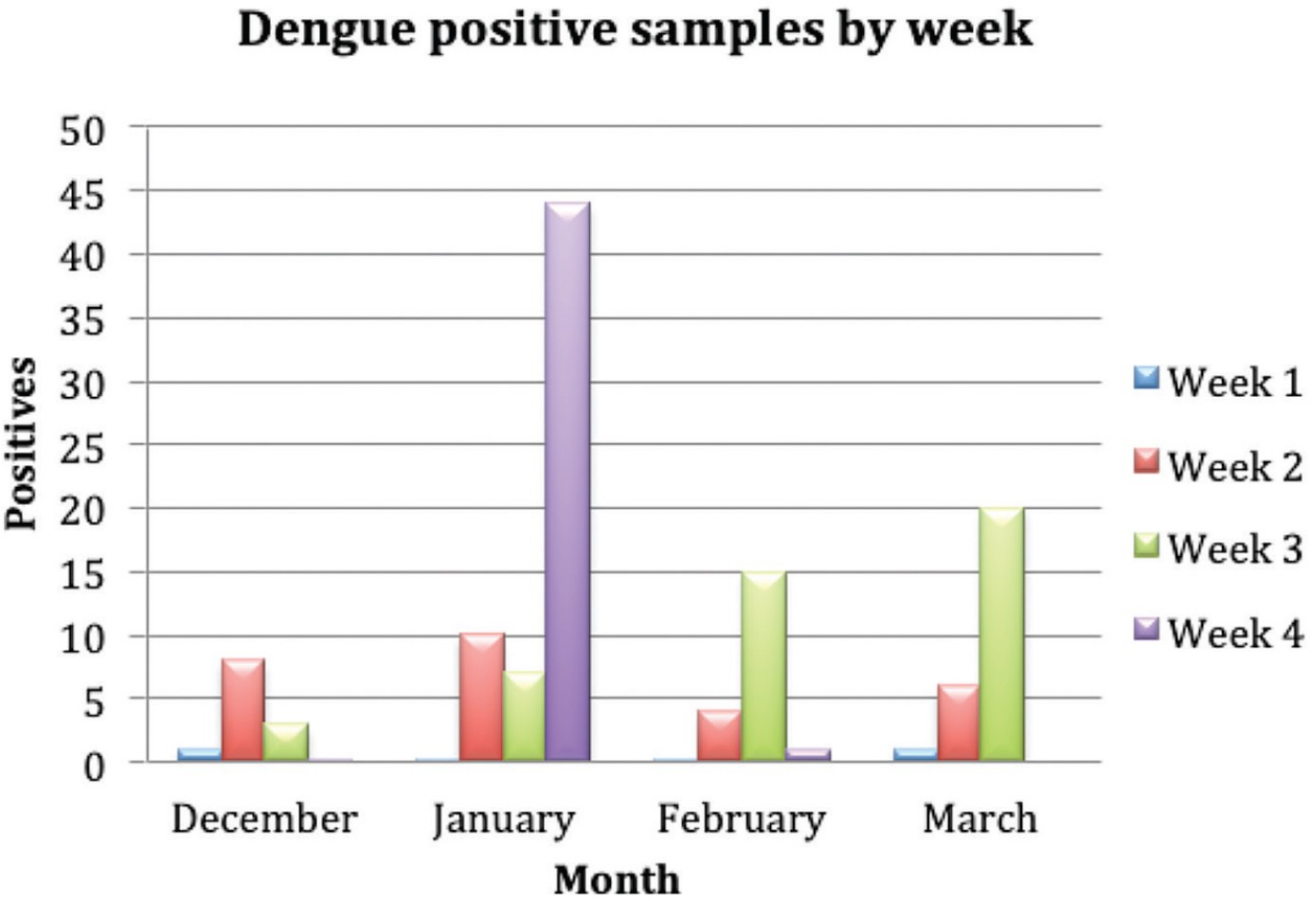
Weekly positive results from December to the 24^th^ March 2017.

No molecular tests were available at Port Vila Central hospital for the detection of dengue virus. Thus samples previously tested for NS1 antigen/IgG and IgM were obtained from patients in the months December 2016 to February 2017. From March 2017 patients attending the hospital with suspected dengue infection were screened with the pan-flavivirus/pan-alphavirus/pan-dengue/dengue-2 assays. Fig 5 shows the positivity by week from December 2016 to March 24^th^ 2017. Molecular testing was carried out between the 12^th^ to the 24^th^ of March.

**Fig 6:**
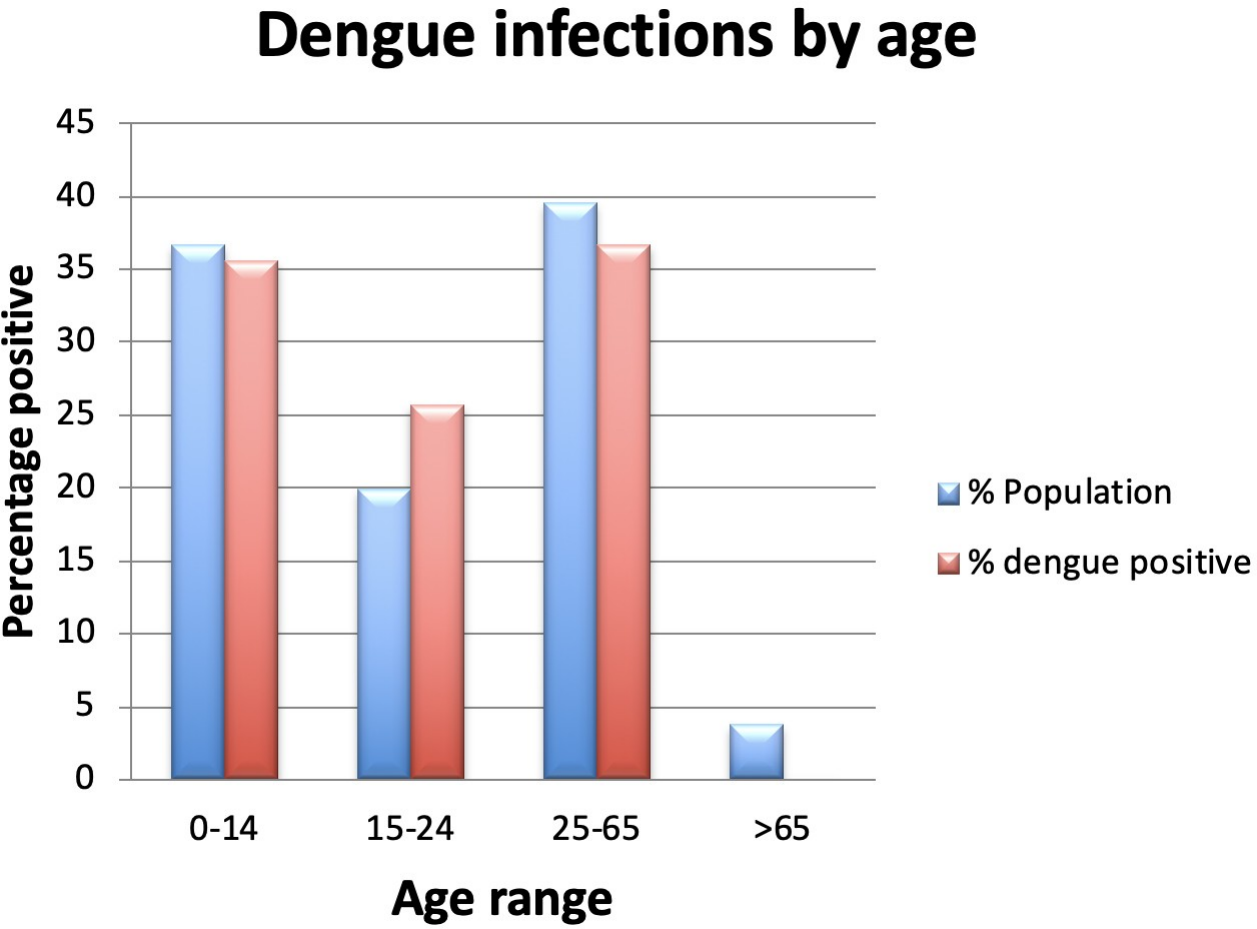
Age distribution of positive samples.

Fig 6 shows the number of dengue infections by age and the age of the general population. As can be seen from the figure the number of dengue positive samples mirrors the age of the population.

## Discussion

The results generated have shown that the 3base assay is able to sensitively and specifically detect the presence of flavivirus, alphavirus and dengue using synthetic constructs, whole viral RNA, whole viral particles, contrived clinical samples as well as validation panels. After initial assay validation the method was then used to test clinical samples obtained during the 2016/2017 dengue 2 outbreak on Vanuatu.

Traditional detection of flaviviruses and alphaviruses in clinical samples has largely relied on Enzyme Immuno Assays. It is well established that EIA assays for ZIKV virus show a high degree of cross reactivity with dengue positive samples [26]. In addition, it has been shown that dengue IgG and IgM assays can cross react with ZIKV-positive patient samples [27]. Furthermore, conventional Enzyme Immuno Assays are unable to differentiate between the individual DENV serotypes and are generally less sensitive than molecular assays. Other methods of detecting unknown aetiological agents of disease include the use of microarrays fabricated with large numbers of oligonucleotides probes specific for individual pathogens. Hybridisation of clinical samples to such arrays can be used to detect the presence of viral genomes in infected individuals [28]. In addition, with the advent of next generation sequencing and the continuing reduction of costs associated with this technology it is now feasible to apply this method to viral discovery [29]. However, these new methods are still more costly, time consuming and lack the sensitivity when compared to more established methods such as RT-PCR, which can generate positive signals in around 1 hour.

The main advantages of molecular testing include the lack of cross reactivity with closely related species and improved sensitivity of pathogen detection. RT-PCR assays can be designed to specifically target individual serotypes. However, very few assays are able to target all the members of complex groups such as flavivirus or alphavirus using a single primer and probe set, without producing PCR bias due to mismatches in the primer or probe sites. In such cases conventional PCR assays require the use of multiple primer and probe sets to detect each virus individually.

In addition, both flavivirus and alphaviruses show a different global distribution with viruses such as YFV endemic in Africa, TBEV endemic in Eurasia, JEV in Southeast Asia, MVEV in Australia and Papua New Guinea, and SLEV mainly found in the United States and more rarely in Canada and Mexico. Similar geographical patterns are also seen with alphaviruses. One advantage of the current pan-flavivirus/pan-alphavirus/pan-dengue screening test is that the assays can be used in any global region to quickly detect the presence of an unknown viral infection. Once a viral family has been identified, individual typing reactions can be used to determine the pathogen responsible for disease. This simplifies and reduces the costs of broad screening approaches in disease outbreaks or during pathogen surveillance in humans or vectors.

The pan-species assays have been shown to amplify any member of the groups using a single primer and probe set. Using synthetic constructs, the pan-alphavirus assay was able to detect CHIKV, WEEV, EEEV, VEEV, RRV, NV, BFV and NV with a linear range 1E9 to 100 and to at least 25 copies per reaction for CHIKV, EEEV, VEEV, RRV, NV, BFV and NV and down to 10 copies per reaction for most viruses (Fig 3). The pan-flavivirus assay was able to detect DENV-1, DENV-2, DENV-3, DENV-4, ZIKV, YFV, WNV and TBEV down to 13 copies per reaction, which was the lowest dilution tested using these constructs (Fig 4).

Contrived clinical samples were prepared by spiking either serum or urine with whole virus particles and processing to determine if these sample types were detrimental to assay performance compared to processing viral particles in molecular grade water only. No significant difference was observed as determined by the Ct values of the EPC or the pan-flavivirus, pan-alphavirus and pan-dengue assays, indicating that the assays were compatible with the routine clinical sample types used for flavivirus and alphavirus molecular testing.

LLOD studies were conducted using whole intact viral particles that had been inactivated in order to render them non-infectious. The results shown demonstrate that when DENV-1, DENV-2, DENV-3, DENV-4, ZIKV and CHIKV inactivated whole virus particles were pooled in a serum matrix, the sensitivity of each assay was around 1–2 copy per reaction for the pan-flavivirus, pan-alphavirus and pan-dengue assays. Using RNA spiked into serum the sensitivity was less possibly due to some degradation of the RNA in the matrix.

No cross reactivity was observed with any component of the assays using a wide range of viral material including adenovirus, BK virus, coronavirus, CMV, enterovirus 68, enterovirus 71, Epstein-Barr virus, Herpes Simplex virus 1, Herpes Simplex virus 2, HHV-6, influenza H1, influenza H3, influenza B, parainfluenza-1, parainfluenza-2, parainfluenza-3, parainfluenza-4A, Respiratory Syncytial virus (subtype A), Respiratory Syncytial virus (subtype B), rhinovirus and Varicella Zoster virus. Positive and extraction controls gave the desired signals thus confirming the negative results.

QCMD panels for DENV, ZIKV virus and CHIKV from 2016–2018 were obtained to monitor the performance of the pan-flavivirus/pan-alphavirus/pan-dengue assays using international standards and reference material. As can be seen from Table 4, results obtained using the pan-species assays and individual typing primers and probes demonstrated 100% concordance with the expected results after sample decoding. In addition, all “educational samples” that were detected by 52.9% to 86.1% of participating laboratories were positive when using the pan-flavivirus/pan-alphavirus/pan-dengue assays. These results indicate that the assays are performing well, if not better than other molecular assays used worldwide.

The Pacific Islands suffer from a high burden of mosquito-borne disease. It was reported that between 2012 and 2014, dengue (serotypes 1–4), CHIKV and ZIKV viruses were circulating in the region with over 120,000 people infected and that this number may be highly underreported. During that time 28 outbreaks of disease were reported. These numbers include 18 DENV (serotypes 1–4), 7 CHIKV and 3 ZIKV outbreaks [30]. These outbreaks result in a heavy toll on the islands’ health system. Thus rapid testing methods are required in the region to enhance surveillance and assist with critical response measures to limit the spread of disease.

Vanuatu is a Pacific Island nation located in the South Pacific Ocean with 83 islands, some of which are sparsely populated, that are home to a population of around 270,000 [31]. Although a number of dengue outbreaks have occurred on the islands the case fatality from dengue has dropped from 6.62/100 in 2002 to 0/100 in 2010 [32]. This reduction is primarily due to increased public awareness for early detection and management. Furthermore, there have been no Chickungunya and only one Zika case recorded on the islands (Kalkie Sero, personal communication). There are 2 main referral hospitals in the region; Port Vila Central Hospital situated in the capital Port Vila, and the Northern District Hospital, Luganville. Port Vila Central is the largest referral hospital in the region and was where molecular testing was performed.

Although the Port Vila Central Hospital was equipped with molecular diagnostic facilities, at the time of this study it did not have access to molecular methods for the detection of flavivirus/alphavirus. Testing carried out included NS1 antigen testing, IgG and IgM serological tests. In order to confirm the presence of DENV samples had to be sent to New Zealand in order to type the strain responsible by molecular diagnostic techniques. This could result in a delay of 2 weeks to confirm the presence and serotype of dengue responsible for infection.

Methods such as real time RT–PCR or nested RT–PCR are widely used to detect DENV genes in acute-phase serum samples. This detection coincides with the viremia and the febrile phase of illness onset [33]. RT–PCR has the additional advantage in that dengue serotype information can be quickly determined, which can assist in patient management, especially in those who go on to develop severe dengue illness. In order to assess the utility of the pan-flavivirus/pan-alphavirus/pan-dengue assay in an outbreak situation, a small footprint (60cm x 60cm x 60cm) cartridge based automated nucleic acid extraction platform (GS-mini, Genetic Signatures, Australia) was used. The GS-mini purification system can process up to 12 samples in a single run in just under 1 hour. The purification platform was simple to use and staff with no prior molecular experience were competent with the process after undergoing only minimal training.

PCR was performed on a MIC real time PCR platform (Bio molecular systems, Sydney, Australia) with a footprint of 13cm x 15cm x 15cm. The MIC is a 48 well 4-colour instrument that takes around 2 hours to complete a run using the pan-flavivirus/pan-alphavirus/pan-dengue assays. Thus 12 individual patient samples from the GS-mini can be processed in a single run using the pan-flavivirus/pan-alphavirus/pan-dengue/DENV-2 assays.

Total turn-around time from sample to result is in the order of 3 hours 30 minutes thus patients can be informed of the results within half a day. In addition, the testing algorithm inspires a high degree of confidence in that three markers are required for a positive result: pan-flavivirus positive; pan-dengue virus positive; and in the case of this particular outbreak, DENV-2 positive. Furthermore, no subsequent confirmation was required thus removing the lengthy process of sending samples to New Zealand for typing.

From the 12^th^ to 24^th^ March 2017 samples from patients with suspected dengue infection were screened using the pan-flavivirus/pan-alphavirus/pan-dengue assay. The sample cohort included archived samples and fresh serum samples from patients attending Port Vila Central Hospital with suspected DENV infection. Prior to testing, a preliminary study was undertaken to determine if samples such as a mouth swab or urine could be used to detect the presence of DENV, instead of the more invasive blood test. In a small number of patients presenting with DENV-like symptoms (n = 4) matched mouth swabs, urine and serum or plasma samples were obtained and processed. Although DENV RNA could be detected in both urine and mouth swabs the levels were significantly reduced compared to the matched serum/plasma samples. Due to the increased sensitivity obtained using serum/plasma samples this sample type was used in all subsequent patient testing.

In total, 187 patient samples with suspected DENV infection were processed using the system (Table 5). Of these, 116 gave triple pan-flavivirus, pan-dengue virus and DENV-2 positive results (62%). There were 7 samples that were positive only for the pan-dengue assay however all these samples gave very weak PCR signals with Ct values greater than 40. Thus DENV RNA may have been present below the LLOD of the assay, and unable to be reliably detected in serum samples. Due to time constraints it was not possible to return to these samples for further confirmation.

When looking at the patient demographics in more detail (where data was available) it was found that there were 61 female cases and 59 male cases of DENV infection in the patient cohort. Anker and Arima previously found an excess of males among reported DENV cases ≥ 15 years of age [34] however this was not supported in this limited study perhaps due to the small sample set.

Fig 5 shows the weekly prevalence of DENV infection from December 2016 to March 24^th^ 2017. The highest numbers of dengue cases were reported in January 2017 with a decline in February. Interestingly, the number of cases of dengue infection then increased again in the month of March. March coincides with the start of routine testing of patients attending the Port Vila Central Hospital with DENV like symptoms using the pan-flavivirus/pan-alphavirus/pan-dengue molecular assays. The increase of dengue cases seen in March may be attributed to an increase in sensitivity when using molecular methods compared to conventional EIA.

Fig 6 shows age related DENV infections. As can be seen from the data the number of positive dengue cases mirrors the age demographics of the islands of Vanuatu [35].

**Fig 7:**
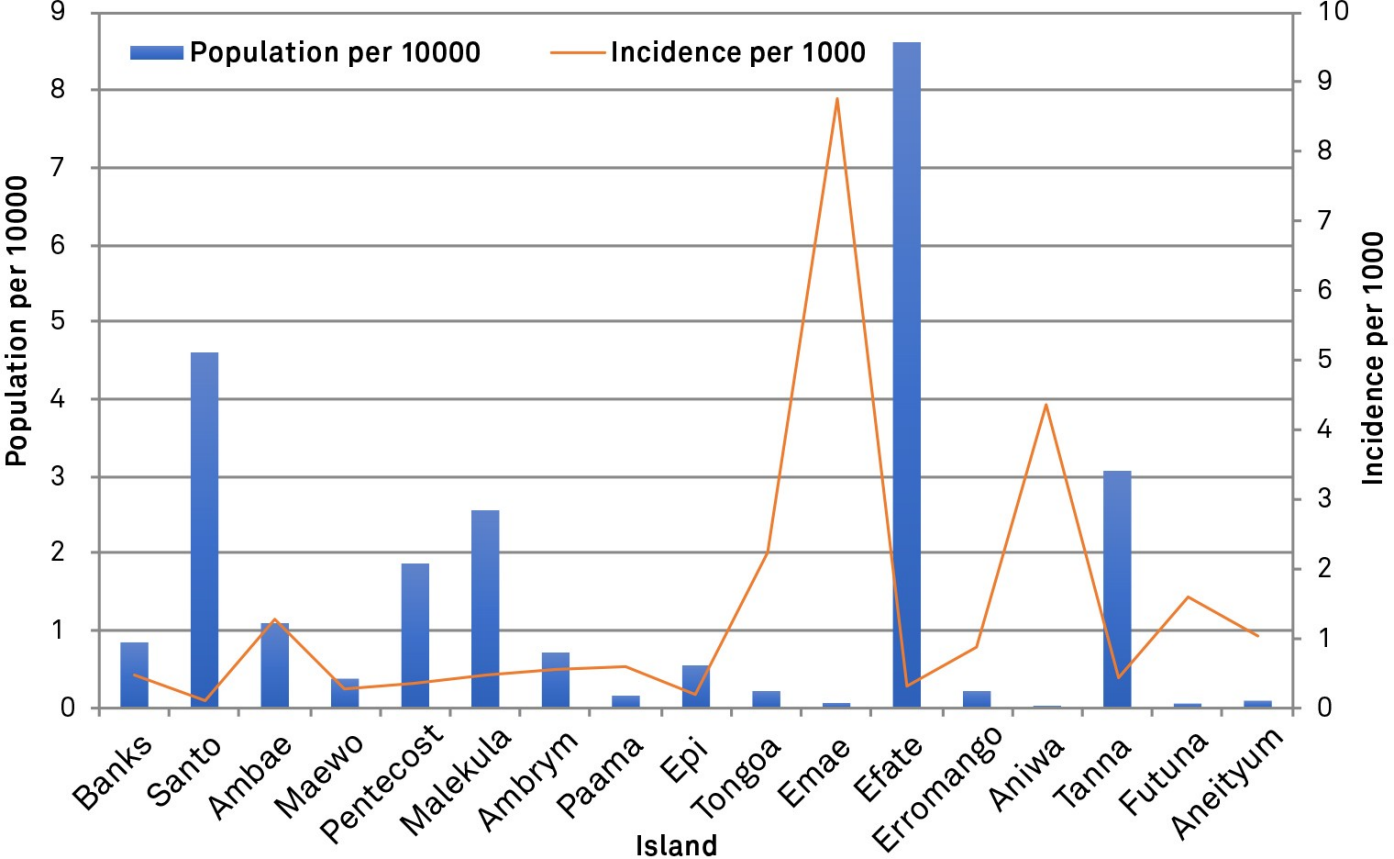
Dengue positive samples per 1000. The figure shows the population of each island in Vanuatu with the number of cases per 1000 island population included.

Fig 7 shows the number of DENV dengue cases by island of residence with the incidence per 1000 residents included. Efate has the largest population of Vanuatu with 86,250 residents and as would be expected also has the highest number of residents with confirmed DENV infection, with 26 cases over the period of testing. The remainder of the islands have a population of 46,078 to as low as 229 or less. In general, the number of positive DENV cases can be correlated with the island population, where for most islands, the incidence of DENV infection was not significantly different to the average national incidence (0.416 per 1000 people). However, the islands of Aniwa (*P* = 0.0035), Ambae (*P*<0.00001), Tongoa (*P*<0.00001), and Emae (*P*<0.00001) had a higher number of positive cases than would be expected from the number of residents alone. Interestingly the island of Santo, with a population of 46, 078, showed a statistically significant decrease in the number of dengue cases recorded (0.018 per 1000; *P* = 0.0016) compared to the national average. However, the most striking examples are the islands of Emae, Tongoa and Ambae. Ambae has a population of 11061 with 14 confirmed dengue cases while Tongoa and Emae had 2243 and 684 residents with a total of 5 and 6 cases respectively. Aniwa had 1 case of dengue and is home to only 229 inhabitants thus can be excluded as a reservoir of increased dengue infection due to the small population and only 1 case of infection. Although this study is relatively small with a total of 116 confirmed cases from the whole Vanuatu region, perhaps the islands Emae, Tongoa and Ambae had a higher number of mosquitoes harbouring DENV thus leading to the increased number of positive cases observed in residents of the island (Fig 7).

In summary the pan-flavivirus, pan-alphavirus and pan-dengue assays have a high sensitivity and specificity when using whole viral nucleic acids and intact viral particles. No cross reactivity was observed with a wide range of non-target organisms and the assay performed well, if not better than other assays used in laboratories worldwide when tested on confirmed reference materials and international standards (QCMD). The pan-flavivirus, pan-alphavirus, pan-dengue virus PCR works well in an outbreak situation and triple positive signals give extra confidence in results. NS1 and pan-flavivirus were in 100% concordance where data was available. Interestingly there was 1 triple positive, pan-flavivirus/pan-dengue/dengue-2 RT-PCR sample that was NS1 antigen negative possibly due to improved sensitivity of the 3base assay.

Results are obtained in just over 3 hours, enabling patients to receive results on the same day. The automated extraction platform and PCR instrument have a small footprint and the assay is easy to use even for staff with no formal molecular training making it ideal to use in situations with limited resources. Finally, the assays as described would be ideally suited as general screening tools or for vector surveillance studies, as any flavivirus or alphavirus can be quickly and sensitively detected regardless of the geographical region or viruses endemic in the region.

## Supporting information

S1 Fig An example of data obtained from a matched patient urine/serum and swab sample. (TIF)
