# Supplementary figures and images for: Validation of the easyscreen flavivirus dengue alphavirus detection kit based on 3base amplification technology and its application to the 2016/17 Vanuatu dengue outbreak
Source: PLoS One. 2020 Jan 17;15(1):e0227550. doi: 10.1371/journal.pone.0227550 (PMC6968865; doi:10.1371/journal.pone.0227550)

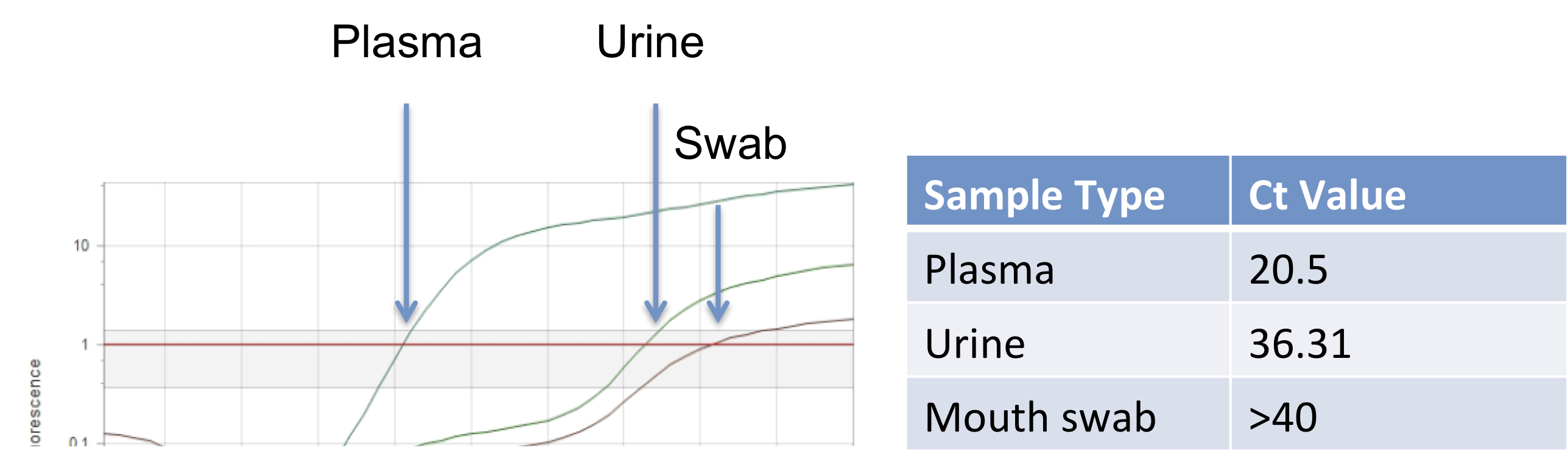

Supplement: S1 Fig — (TIF) [file pone.0227550.s001.tif]
